# Korean *Artemisia* species: phylogenomic authentication, ethnopharmacology, and commercial applications

**DOI:** 10.3389/fphar.2026.1873437

**Published:** 2026-09-16

**Authors:** Adnan Amin, Khizar Abbas, Mozaniel Santana de Oliveira

**Affiliations:** 1 Department of Life Sciences, Yeungnam University, Gyeongsan, Republic of Korea; 2 School of Pharmacy, University College London, London, United Kingdom; 3 Laboratory of Pharmacology of Inflammation and Behavior, Graduate Program in Pharmaceutical Sciences, Institute of Health Sciences, Federal University of Pará, Belém, Brazil

**Keywords:** Aeyup, *Artemisia iwayomogi*, chloroplast genome, ITS2, Korean peninsula, polyphenols

## Abstract

*Artemisia* species are integral to traditional Korean medicine and are increasingly used in medicinal and cosmeceutical products. However, taxonomic ambiguity, chemical variability, and limited clinical validation constrain their reliable therapeutic and commercial utilization. This review critically examines how phylogenomic evidence can be integrated with ethnopharmacology and phytochemistry to strengthen the authentication, scientific evaluation, and quality-controlled development of *Artemisia* taxa occurring in Korea. Its distinctive contribution is a Korea-focused synthesis linking chloroplast-genome variation, traditional medicinal categories, species-associated phytochemical profiles, pharmacological evidence, and commercial quality requirements. Complete plastome analyses provide greater phylogenetic resolution than many single-locus approaches, whereas variable regions such as *ycf1*, *accD*, and selected intergenic spacers offer candidate markers for species authentication. Nevertheless, chloroplast-based identification may remain insufficient for closely related taxa and should be complemented by nuclear markers, morphology, and chemical profiling. Korean *Artemisia* taxa contain sesquiterpene lactones, flavonoids, phenolic acids, and volatile terpenoids associated mainly with anti-inflammatory, antioxidant, antimicrobial, and hepatoprotective effects in preclinical studies. These findings should not be interpreted as established clinical efficacy. Plastome divergence alone does not directly explain specialized-metabolite production or therapeutic activity. Future research should prioritize authenticated germplasm, integrated multi-omics, standardized extraction, toxicological and clinical validation, sustainable cultivation, and harmonized regulatory quality control.

## 1 Introduction

The genus *Artemisia* (Asteraceae) has a global distribution in temperate and subtropical regions, with above 500 species (Shinyuy et al., 2023). These species are known for their ecological versatility and widespread use in traditional medicine (Nurlybekova et al., 2022; Hussain et al., 2024). *Artemisia* has been recognized for its diverse medicinal properties, including Antidiabetic, anti-cancer, anti-inflammatory, antimicrobial, antimalarial activities, and Management of G.I.T disorders (Bisht et al., 2021; Ekiert et al., 2022).

The Korean-native *Artemisia* species (above 30) (Kim et al., 2013; Kang et al., 2024) comprises a diverse group that warrants increased attention due to their unique phylogenetic and phytochemical profiles (Amin and Park, 2025; Yong et al., 2025). Among these, Key species, including *A. argyi, A. capillaris, Artemisia iwayomogi*, *Artemisia fukido* Makino, and *A. annua*, have been used for centuries in Korea and surrounding regions for therapeutic purposes (Ekiert et al., 2021; Chen et al., 2024a; Yong et al., 2025). They are well known for key role in treating digestive disorders, enhancing circulation, and mitigating inflammation (Ekiert et al., 2021; Sharifi-Rad et al., 2022; Jin et al., 2025). Several bioactive compounds have been isolated from these species, such as flavonoids, sesquiterpene lactones, and phenolic acids (Hong et al., 2023; Hussain et al., 2024; Park et al., 2024). Several flavonoids (apigenin, quercetin, kaempferol) have been reported from *Artemisia argyi* that possess potent antioxidant and anti-inflammatory properties (Wang et al., 2023a)*.* Likewise, *Artemisia annua* is the principal source of artemisinin, a sesquiterpene lactone with established antimalarial activity (Morua et al., 2025). However, reported activities remain dominated by ethnomedicinal, *in vitro*, and animal evidence. Clinical evidence remains limited for most non-artemisinin preparations. Species identity, chemotype, plant organ, and extraction method strongly influence reported findings. A diverse distribution of secondary metabolites in *Artemisia* spp. from Korean peninsula is further explained by the chloroplast genome (CP genome) analysis that further uncovers inter-species evolutionary relationships (Kim G.-B. et al., 2020). Several, molecular investigations using chloroplast markers such as rbcL, matK, and trnL-F have explained relationships within the genus and its subgenera (Lan et al., 2022). Based on CP genome data, research has confirmed that *Artemisia* species from different geographic regions, including Korean and East Asian species, form “distinct clades”, explaining the role of environmental and evolutionary pressures on phylogeny (Kim G.-B. et al., 2020; Kadam et al., 2024). Based on these parameters from CP genome analysis, *Artemisia littoricola* obtained from Dokdo island (Korea) was placed within subgenus *Dracunculus*, showing a closer relationship with *Artemisia scoparia* and *Artemisia desertorum.* From additional biogeographic reconstructions it was evident that its ancestral origins is in East Asia, especially in Mongolia, China (North East and North Central and South Central China), and Korea (Kadam et al., 2024). The understanding of integrated chemotaxonomic and phylogenetic marker analyses is also helpful in understanding the evolution of *Artemisia* species. For instance, analyses of Korean native *Artemisia gmelinii, Artemisia capillaris,* and *Artemisia fukudo* revealed genomic variations and phylogenetic relationships among these species, in addition to the identification of certain flavonoids, phenolic acids, and terpenoids as potential chemical markers (Lee et al., 2022). A combined approach to understand evolutionary molecular mechanism of *Artemisia* is also popular. In a transcriptome and metabolite profiling based investigation, the gene expression with flavonoid (eupatilin and jaceosidin) synthesis was investigated to correlate genomic variation including chloroplast genes affecting precursor metabolism with metabolic phenotypes (Lee et al., 2023).

Previous reviews summarized genus-wide phytochemistry, pharmacology, toxicology, essential oils, or cosmetic applications. Others examined individual species, particularly *A. annua*, rather than Korean *Artemisia* collectively (Nigam et al., 2019; Ekiert et al., 2021). Korean studies generally address individual taxa, chemotypes, or authentication methods (Lee et al., 2022; Hong et al., 2023). Our search identified no comprehensive Korea-focused synthesis covering these linked themes. This review addresses that gap through a Korea-focused, evidence-based framework. Plastome evidence supports authentication, phylogeny, conservation, and quality control. It does not directly establish therapeutic efficacy. The central question concerns integrating phylogenomic, phytochemical, and ethnopharmacological evidence for Korean *Artemisia*. This review evaluates authentication, medicinal use, pharmacological evidence, conservation, and commercial quality requirements. It also identifies barriers to standardized, safe, and sustainable product development.

## 2 Literature search strategy

Botanical names were verified using Kew’s Medicinal Plant Names Services and Plants of the World Online. Accepted names and authorities were recorded for each taxon. This structured narrative review searched PubMed, Scopus, Web of Science, and Google Scholar. Korean records were additionally identified through RISS, KoreaMed, and the Korean Traditional Knowledge Portal. Searches covered database inception through last 10 years. Search terms combined “Korean *Artemisia*,” “Korean *Artemisia* phylogeny”, “*Artemisia* plastome”, “authentication”, “phytochemistry”, “ethnopharmacology”, “pharmacology”, “safety”, “conservation”, and “commercialization” of Korean Artemisia. Boolean operators were adapted for each database. Records were exported to reference-management software, and duplicates were removed. Titles and abstracts were screened before full-text assessment. Reference lists of eligible studies were examined for additional records. Primary studies addressing Korean *Artemisia* taxonomy, genomics, chemistry, pharmacology, safety, or applications were included. Reviews supported contextual interpretation and reference tracing. Official sources supported taxonomy, regulation, traditional use, and commercial product verification. Duplicates, irrelevant reports, inaccessible texts, and insufficiently identified plant materials were excluded. Evidence was classified as genomic, phytochemical, *in vitro*, *in vivo*, clinical, regulatory, or commercial. Plant-extract studies were appraised using ConPhyMP reporting principles (Heinrich et al., 2022). Findings were synthesized narratively because methods, preparations, models, and outcomes were heterogeneous. No meta-analysis was performed because outcomes lacked sufficient comparability (Frontiers Ethnopharmacology guidelines).

## 3 Botanical and taxonomic framework

The plants of genus *Artemisia* are extremely diverse across various geographic regions (Jiao et al., 2023). Plants of this genus are commonly distributed in subtropical and temperate region and are being used by human since centuries for various health concerns (Jiao et al., 2025). Nevertheless, a clear understanding of global diversity needs a careful and detailed taxonomic analysis including morphological, molecular and ecological data.

### 3.1 Distribution of Korean *Artemisia*

The plants from genus *Artemisia* are best known for aroma and are integral part of Korean food, TKM and agriculture since ancient times (Kim et al., 2022). In Korean peninsula, the *Artemisia* species thrive to grow in different habitats (mountains, grasslands, temperate forests and wetlands) (Kim M.-H. et al., 2020; Kim et al., 2022). These plants are found across distinct geographic regions, with some species preferring high-altitude, colder climates, whereas others are common in warmer, lowland areas (Kolbek et al., 2020; Kim et al., 2022). The following table provides a comprehensive overview of native *Artemisia* species in Korea, their geographic locations, and habitats (Table 1).

**TABLE 1:** Selected *Artemisia* taxa recorded from the Korean Peninsula, with their nomenclatural treatment, distributional status, and documented ecological information.

S.No.	Taxon	Authority	POWO/WCVP treatment	Korean distribution/status	Habitat/ecological information	References
1	*Artemisia annua*	L.	Accepted	Included in the Korean native-flora checklist; POWO records Korea within its native range	Annual plant occurring primarily in the temperate biome	(Son et al., 2025; Plants of the World Online (POWO), 2026)
2	*Artemisia sacrorum* var. *iwayomogi*	(Kitam.) M.S.Park and G.Y.Chung	Synonym of *A. gmelinii* var. *gmelinii* in POWO	Included in the Korean native-flora checklist; voucher-documented in South Korea	Temperate habitats; detailed Korean localities are provided by Park et al., 2016	Park et al., 2016; Son et al., 2025; Son et al., 2025 Plants of the World Online (POWO), 2026)
3	*Artemisia argyi*	H.Lév. and Vaniot	Accepted	Included in the Korean native-flora checklist; POWO records Korea within its native range	Primarily temperate biome	(Son et al., 2025; Plants of the World Online (POWO), 2026)
4	*Artemisia fukudo*	Makino	Accepted	Included in the Korean native-flora checklist; POWO records Korea within its native range	Coastal salt marshes and estuarine habitats; documented on the southwestern coast of South Korea	Son et al., 2025; Plants of the World Online (POWO), 2026
5	*Artemisia capillaris*	Thunb.	Accepted	Included in the Korean native-flora checklist; POWO records Korea within its native range	Temperate biome; also documented in coastal salt-marsh communities of southwestern South Korea	Son et al., 2025; Plants of the World Online (POWO), 2026
6	*Artemisia japonica*	Thunb.	Accepted	Included in the Korean native-flora checklist; POWO records Korea within its native range	Perennial occurring primarily in the temperate biome	Son et al., 2025; Plants of the World Online (POWO), 2026
7	*Artemisia montana*	(Nakai) Pamp.	Accepted	Included in the Korean native-flora checklist; POWO records Korea within its native range	Perennial occurring primarily in the temperate biome	Son et al., 2025; Plants of the World Online (POWO), 2026
8	*Artemisia selengensis*	Turcz. ex Besser	Accepted	Included in the Korean native-flora checklist; POWO records Korea within its native range	Perennial or rhizomatous geophyte of the temperate biome	Son et al., 2025; Plants of the World Online (POWO), 2026
9	*Artemisia scoparia*	Waldst. and Kit.	Accepted species; POWO classifies its Korean occurrence as introduced	Included as native in the Korean checklist, although POWO records it as introduced in Korea	Temperate biome; documented from disturbed coastal salt-marsh and beach environments in South Korea	Son et al., 2025; Plants of the World Online (POWO), 2026
10	*Artemisia littoricola*	Kitam.	Accepted species; Korea is omitted from the current POWO range	Included in the Korean native-flora checklist and molecularly documented from Dokdo Island	Temperate island occurrence; the cited genome study does not provide a detailed habitat assessment	(Kadam et al., 2024; Son et al., 2025; Plants of the World Online (POWO), 2026
11	*Artemisia caruifolia*	Buch.-Ham. ex Roxb.	Accepted species; *A. apiacea* is a synonym of *A. caruifolia* var. *caruifolia*	Included in the Korean native-flora checklist; POWO records Korea within its native range	Perennial occurring primarily in subalpine or subarctic biomes	Son et al., 2025; Plants of the World Online (POWO), 2026
12	*Artemisia sacrorum*	Ledeb.	Synonym of *A. gmelinii* var. *messerschmidtiana* in POWO	Retained in the Korean native-flora checklist under the Korean regional treatment	Temperate biome under the corresponding POWO treatment	(Park et al., 2016; Son et al., 2025; Plants of the World Online (POWO), 2026
13	*Artemisia stolonifera*	(Maxim.) Kom.	Synonym of A. argyi var. argyi in POWO	Retained in the Korean native-flora checklist	Temperate biome under the corresponding POWO treatment	Son et al., 2025; Plants of the World Online (POWO), 2026
14	*Artemisia keiskeana*	Miq.	Accepted	Included in the Korean native-flora checklist; POWO records Korea within its native range	Primarily temperate biome	Son et al., 2025; Plants of the World Online (POWO), 2026
15	*Artemisia indica*	Willd.	Accepted	Included in the Korean native-flora checklist; POWO records Korea within its native range	Perennial occurring primarily in the temperate biome	Son et al., 2025; Plants of the World Online (POWO), 2026
16	*Artemisia rubripes*	Nakai	Accepted	Included in the Korean native-flora checklist; POWO records Korea within its native range	Perennial occurring primarily in the temperate biome	Son et al., 2025; Plants of the World Online (POWO), 2026
17	*Artemisia sieversiana*	Ehrh. ex Willd.	Accepted species; *A. koreana* is treated as its synonym	Included in the Korean native-flora checklist; POWO records Korea within its native range	Annual or biennial occurring primarily in the temperate biome	Son et al., 2025; Plants of the World Online (POWO), 2026
18	*Artemisia lancea*	Vaniot	Accepted species; *A. feddei* is treated as its synonym	Included in the Korean native-flora checklist; POWO records Korea within its native range	Perennial occurring primarily in the temperate biome	Son et al., 2025; Plants of the World Online (POWO), 2026
19	*Artemisia hallaisanensis*	Nakai	Synonym of *A. capillaris* in POWO	Retained as a Korean-endemic taxon in the Korean checklist; documented from Jeju Island	Jeju Island occurrence; precise habitat was not characterized in the cited plastome study	Lim et al., 2018; Son et al., 2025; Plants of the World Online (POWO), 2026

Taxonomic names used for the Korean flora follow Son et al. (2025). Nomenclatural authorities and global taxonomic treatments were cross-checked against IPNI, and POWO/WCVP., Where the Korean checklist and POWO/WCVP, apply different treatments, both are reported. Habitat information derived from POWO, represents the broad global biome and should not be interpreted as a detailed habitat assessment for Korean populations.

### 3.2 Morphological diversity of Korean *Artemisia* species

Korean *Artemisia* taxa vary substantially in leaves, trichomes, inflorescences, and growth forms. These traits support identification but may reflect phenotypic plasticity and convergent evolution (Bora and Sharma, 2011). In Korea, more than 30 *Artemisia* species have been reported, including the most-studied species vis *A*. *argyi*, *A*. *annua*, and *A*. *iwayomogi*, each with unique morphological traits that are integral to their identification, classification, and ecological roles (Sung et al., 2008).

*Artemisia argyi* has deeply lobed leaves with densely pubescent abaxial surfaces (Cui et al., 2022; Chen et al., 2024a). Conversely, *A. annua* has narrower, more dissected, and less pubescent leaves (Wetzstein et al., 2014). These differences support field identification but require molecular confirmation under variable environments.

The essential oils from *Artemisia* species (terpenoids) are mainly synthesized and stored in these trichomes (Janaćković et al., 2019). In *A*. *argyi*, trichomes (glandular and nonglandular) are particularly abundant on the undersides of the leaves with the high levels of essential oils utilized in traditional medicine (Cui et al., 2022). On the other hand, the *A. iwayomogi* possesses no non-glandular trichome on both leaf surfaces (Park et al., 2016), whereas *A. annua* possesses glandular trichomes as the primary artemisinin biosynthesis site (Wang et al., 2009). Glandular trichomes are specialized sites for terpenoid biosynthesis and storage. In *A. annua*, they support artemisinin biosynthesis. In *A. argyi*, non-glandular trichomes also contribute to moxa quality. However, trichome density alone cannot predict metabolite concentration without chemical validation (Zhang et al., 2024; Zhao et al., 2024). In *A*. *argyi* however, fewer trichomes are noticed on upper surface (adaxial) of the leaves lower surface (abaxial), although glandular trichomes are also present (Lee et al., 2023).

Floral morphology varies among Korean *Artemisia* taxa and supports preliminary species identification. *Artemisia annua* bears small capitula arranged within branched terminal panicles. Each capitulum contains marginal pistillate florets and central bisexual disk florets. Disk florets are protandrous, while earlier pistillate flowering promotes outcrossing. These features support breeding and controlled seed production in *A. annua* (Wetzstein et al., 2014). *Artemisia argyi* also shows diagnostic inflorescence and capitulum morphology (Lee J. H. et al., 2018). Taxonomic descriptions likewise recognize capitulum traits in *A. iwayomogi* (Park et al., 2016). However, reported floret counts and pollination modes require species-specific experimental confirmation. Capitulum forms evolved repeatedly across *Artemisia*, generating substantial morphological homoplasy (Jiao et al., 2023). Therefore, floral traits should complement molecular evidence during taxonomic and evolutionary inference.

Korean taxa include annual and perennial herbs, with occasional semi-woody growth forms (Nataraj et al., 2022). Growth-form variation may support persistence across contrasting lowland and montane habitats. However, these adaptive relationships require population-level ecological validation (Abbas et al., 2016; Chaney et al., 2017). Morphology remains valuable for preliminary identification and medicinal-material authentication. However, environmental plasticity can obscure species boundaries (Park et al., 2016; He et al., 2024). Morphological evidence should therefore complement genomic authentication and metabolite profiling (Figure 1).

**FIGURE 1:**
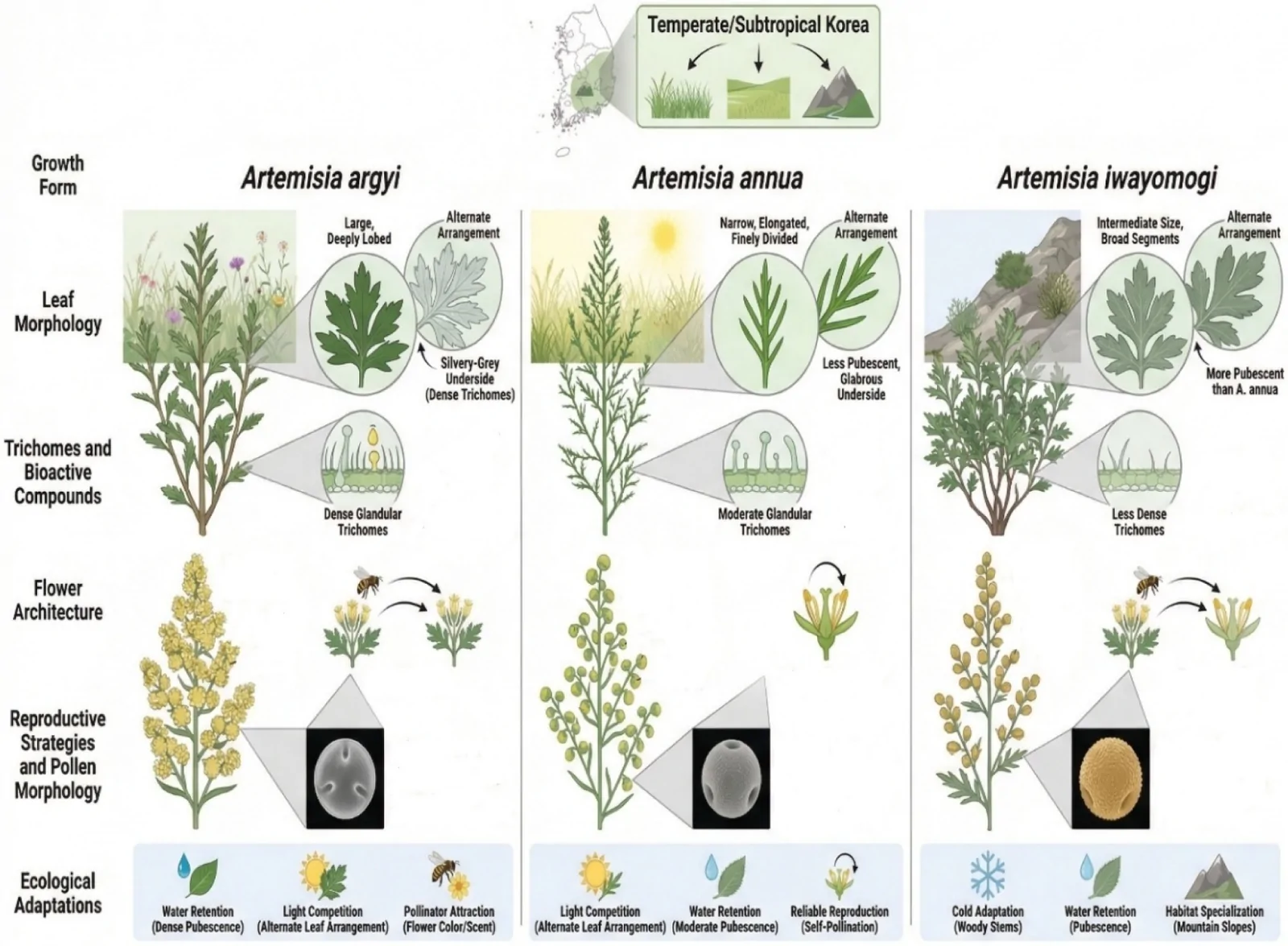
Comparative morphology of selected Korean *Artemisia* taxa. Panels summarize leaf form, trichomes, inflorescences, growth habit, and root architecture. Illustrations are schematic and not drawn to scale. These traits support preliminary identification but require molecular confirmation.

### 3.3 Conservation status

Authoritative floristic and taxonomic sources record *A. annua, A. argyi, A. capillaris,* and *Artemisia selengensis* as native to Korea, while *Artemisia princeps* is also reported from Korea in regional floristic literature; nevertheless, these occurrence records alone do not establish their population abundance or national conservation status (Son et al., 2025; Son et al., 2025; POWO, 2026). *Artemisia hallaisanensis* is treated as an endemic Jeju taxon in Korean literature, although POWO currently places this name in synonymy with *A. capillaris*, indicating unresolved taxonomic circumscription (Lim et al., 2018; Chung et al., 2023). *Artemisia fukudo* occurs in Korean coastal salt-marsh and estuarine habitats but has a broader East Asian distribution (Lee S. H. et al., 2018; Kang et al., 2024). Similarly, *Artemisia littoricola* has been documented from Dokdo Island, but it is not Korean-endemic and occurs elsewhere in East Asia (Kadam et al., 2024). No species-level global or Korean Red List category was located for these focal taxa in the sources consulted; therefore, no formal threat status is assigned here. Targeted population surveys and habitat-specific monitoring are required before conservation priorities, including *ex situ* measures, can be established.

Among other practical strategies, the establishment of “protected areas for *Artemisia* spp.” is one promising conservation strategy, where *Artemisia* species can thrive in their natural habitats without human interference (Scott et al., 2010). Through sustained application of this strategy, for commercial *Artemisia* harvesting, pressure on wild populations can be alleviated (Yuliani, 2025). Furthermore, such an initiative (conservation) can keenly assist in maintaining the ecological balance by ensuring that these valuable plants are available for medicinal use while preserving biodiversity. Another important strategy includes cultivation of native species of *Artemisia* for commercial applications. It is very helpful in alleviating the impact on wild populations and contribute to the long-term sustainability (Chen et al., 2016). Lastly, mostly adopted strategy is integration of *in-situ* and *ex-situ* conservation strategies. Such strategies are considered crucial for preserving agriculturally important *Artemisia* species and maintaining their medicinal and ecological roles (Zegeye, 2017).

Molecular authentication represents important progress beyond morphology-based identification. However, phenotypic plasticity and limited barcode variation still obscure several species boundaries. Population sampling, verified vouchers, and common-garden studies should test disputed taxonomic assignments (Jiao et al., 2025).

## 4 Phylogenetic insights and chloroplast genome evolution

One of the most important concerns in *Artemisia* species living in Korea is “misidentifications” that exist due to high morphological variability (Jiao et al., 2025). However, in modern times, employment of DNA barcoding and CP genome analysis using markers like rbcL, matK, and ITS has removed this limitation. Below is a detailed overview of CP genome analysis of commercially important Korean *Artemisia* species.

### 4.1 CP genomes as evolutionary markers in Korean *Artemisia* spp

The phylogenetics analysis using CP genome is based on conserved and variable sequences that are typically provided by its quadripartite structure and key genes including the LSC, SSC, and two IR regions (Zhang et al., 2023). In *A*. *annua*, the detailed comparative evolutionary analysis across species mainly depends on the CP genome that is 150,995 bp long, with IR regions flanking single copy regions (Shen et al., 2017). Generally, the chloroplast gene contents are comprised of ∼113 unique genes, along with highly conserved genes of photosynthesis and genetic machinery (Daniell et al., 2016). Yet the divergence is noticed in variable intergenic regions and specific loci including ycf1, accD, and rps19, making them effective phylogenetic markers (Guo et al., 2025). Key features of the CP genome, comprising a comparatively slower mutational rate and an overall lack of recombination and reduced homoplasy, make it very useful in understanding evolutionary history (Wang et al., 2024). Thus, chloroplast genomes provide both resolution and reliability for evolutionary inference across *Artemisia* lineages.

In modern times, the whole chloroplast sequencing greatly used compared to old single locus approaches involving rbcL or matK, since it offers robust resolution of complicated phylogenetic relationships among species (Leaks et al., 2025). Genome-wide phylogenetic reconstructions using entire plastomes or core protein-coding gene sets provide higher resolution and better support values than single-gene analyses (Kim G.-B. et al., 2020). For example, complete plastome analyses can discriminate among closely related *Artemisia* species that were ambiguous under ITS or psbA-trnH markers (Chen et al., 2024b; Guo et al., 2025). Complete plastomes often improve phylogenetic resolution among closely related taxa. However, plastomes trace one inherited lineage and may overlook reticulate evolution. Hybridization, introgression, and limited divergence can further reduce discriminatory power. Early complete plastome sequencing work includes Korean-used medicinal mugworts such as *A*. *argyi* (“Aeyup” in Korea). This study reported plastome size, gene content, and high sequence identity with close congeners that are useful for both phylogeny and authentication (Kang et al., 2016). Similarly, another key study characterized the full chloroplast genomes of *A. capillaris and Artemisia gmelinii* and used them to assess relationships in the genus, explicitly positioning plastomes as a basis for barcoding and authentication in *Artemisia* (Lee et al., 2016).

### 4.2 Comparative plastome analyses of *Artemisia* species

Comparative plastome analyses reveal evolutionary patterns by examining structure, content, and sequence divergence across Korean *Artemisia* species. Complete plastome comparisons for five *Artemisia species, including Artemisia lactiflora, A. princeps, Artemisia indica, A. argyi and Artemisia lancea,* demonstrated a typical quadripartite structure and were highly conserved across species with varying lengths (151,132–151,178 bp), and their gene content and codon preferences were similar. Mutation hotspot analysis identified four highly variable regions, including rpl32_trnL-UAG, trnY-GUA_trnE-UUC, ndhH_rps15, and ycf1*.* It was evident that these species form sister clades, and individuals of each species form a monophyletic clade (Lan et al., 2022).

The comparative plastome analysis is also very helpful to clarify detail about *Artemisia* evolution and phylogenomics. This is based on the fact that single-locus cpDNA (e.g., *trnL–F*) often lacks resolution among closely related *Artemisia*. With this context, the development of PCR-friendly InDel barcodes gives a lankmark “translational” output of comparative plastome analysis. A clear example is the medicinal-herb authentication analysis included that assembly of multiple plastomes in *A. gmelinii*, *A. capillaris*, and *A. fukudo* species. This was further extended by designing seven InDel-based barcode markers from polymorphic regions (including regions involving *ycf1* and multiple intergenic spacers) (Lee et al., 2022). In another CP genome based investigation, assembly and comparative plastome analysis of 38 *Artemisia annua* was performed. With observation of several distinct intraspecific divergences, a clear discrimination among species (artemisinin production) was recorded (Ding et al., 2024).

### 4.3 Phylogenetic relationships among Korean *Artemisia* species

Global phylogenomic work confirms that many traditional infrageneric groupings based on morphology are not strictly monophyletic and that higher-resolution genomic datasets are needed to stabilize relationships (Jiao et al., 2023). Jiao et al. (2025) further demonstrated extensive cytonuclear discordance in *Artemisia*, potentially reflecting rapid diversification, incomplete lineage sorting, hybridization, and chloroplast capture. Therefore, plastome phylogenies should be interpreted as hypotheses of plastid-lineage history rather than definitive species trees and should be integrated with low-copy nuclear genes, nrDNA, morphological traits, and biogeographic evidence. Chloroplast data remain valuable for authentication and marker discovery, but robust evolutionary conclusions require cytonuclear integration.

In Case of Korean perspective, used three tiers of data including Chloroplast trnL–F, Nuclear ribosomal ITS and CP based analysis with each giving a different level of phylogenetic clarity. The Chloroplast trnL–F based investigation A widely cited Korean authentication study tested 21 Korean *Artemisia* species with cpDNA trnL–F PCR-RFLP and also analyzed trnL–F sequences. The key phylogenetic takeaway was that trnL–F has insufficient variation to reliably discriminate most Korean species many taxa collapse into a small number of banding/sequence groups. However, it still produced species-specific patterns for a few taxa (notably *Artemisia apiacea, Artemisia keiskeana, and Artemisia sieversiana*) that can be used as diagnostic DNA markers (Lee et al., 2009). A dedicated Korean phylogenetic study analyzed nrDNA ITS sequences across Korean *Artemisia* taxa to reconstruct relationships and found ITS to be useful for Korean mugwort systematics (compared to low-variation cp markers) (Lee et al., 2010). Because ITS is nuclear and typically more variable, it often separates closely related taxa better than trnL–F. This is also consistent with broader medicinal-plant authentication literature that uses ITS/ITS2 for discriminating *Artemisia* materials (Doh et al., 2016). The Plastome-scale phylogenies have transformed *Artemisia* systematics because they provide thousands of informative sites (far more than trnL–F). East Asian plastome comparisons show that whole plastomes can separate many taxa and recover major clades/subgenera, while also revealing sets of species whose plastomes are nearly indistinguishable (Kim G.-B. et al., 2020). For Korean-relevant taxa, plastome resources include Complete plastomes of *A. gmelinii* and *A. capillaris* (both used as herbal medicine in Korea), placed within a broader *Artemisia* plastome phylogeny (Lee et al., 2022) and comparative plastome work among *A. gmelinii, A. capillaris, and A. fukudo* that quantifies inter- and intraspecific divergence and develops diagnostic markers from plastome hotspots (Jiao et al., 2025). To summarize the published phylogenetic evidence, a simplified cladogram of representative Korean *Artemisia* taxa is presented in Supplementary Figure S1 (Supplementary Material).

### 4.4 Comparative utility and translational relevance

Marker performance varies with taxonomic depth, sample quality, and authentication purpose. The *rbcL* marker amplifies reliably but often lacks species-level resolution. *matK* offers greater variation, although amplification success can be inconsistent. The *trnL–F* region provides limited discrimination among closely related Korean taxa. Nuclear ITS offers higher variability but may reflect paralogy or hybridization (Lee et al., 2009; Zhu et al., 2022).

Complete plastomes usually provide stronger resolution and reveal candidate diagnostic regions. These include *ycf1*, *accD*, *rpl32–trnL*, and several intergenic spacers. However, candidate loci require population-level validation before regulatory adoption (Kim et al., 2020; Chen et al., 2024b). Therefore, no single marker provides universal authentication across Korean *Artemisia*.

A tiered framework offers stronger translational value for herbal regulation. Standard barcodes can support initial screening of intact botanical materials. Validated hotspot markers can distinguish selected substitutes and closely related taxa. Whole plastomes can resolve difficult samples and strengthen curated reference databases. Mini-barcoding supports processed products containing degraded DNA. Metabarcoding is preferable for products containing multiple species. Chemical profiling must confirm identity, potency, and batch consistency. Voucher specimens and validated sequences remain essential for regulatory traceability (Lee et al., 2022; Mahima et al., 2022) (Table 2).

**TABLE 2:** Comparative utility of molecular approaches for *Artemisia* authentication.

Marker	Main strength	Main limitation	Application	References
*rbcL*	Reliable amplification and sequencing	Low species resolution in *Artemisia*	Initial screening with another locus	(Hollingsworth et al., 2009)
*matK*	Standard plant barcode with moderate variation	Variable recovery and weak *Artemisia* resolution	Combined *rbcL–matK* screening	(Liu et al., 2017)
*trnL–F*	Simple PCR–RFLP workflow	Resolved only three of 21 Korean taxa	Targeted Korean species authentication	(Lee et al., 2009)
ITS or ITS2	High resolution in tested *Artemisia* taxa	Multicopy variation and contamination risks	Closely related taxa and *A. argyi*	(Mei et al., 2016)
Plastome hotspots and InDels	High local sequence variability	Validation remains taxon-specific	Targeted assays after validation	(Lee et al., 2009)
Complete plastome	High phylogenetic information	Closely related taxa may remain unresolved	References phylogeny and marker discovery	(Chen et al., 2024b)
Mini-barcoding	Works with degraded DNA	Short fragments reduce resolution	Processed single-ingredient products	(Parveen et al., 2016)
DNA metabarcoding	Detects multiple taxa simultaneously	Primer and database biases	Mixed or polyherbal products	(Raclariu et al., 2017)
DNA and chemical profiling	Confirms identity and chemical composition	Higher cost and analytical demand	Orthogonal quality control	(Lee et al., 2022)

### 4.5 Molecular evolutionary patterns

Molecular evolutionary patterns within *Artemisia* chloroplast genomes reflect a balance between structural conservation and localized divergence driven by ecological adaptation.

Key comparative plastome studies on *Artemisia* consistently show that chloroplast genomes are structurally conserved (with a typical LSC–SSC–IR organization) but exhibit concentrated sequence divergence in a limited set of hotspot regions, enabling higher phylogenetic resolution than single-locus cpDNA. A large East Asian plastome dataset (≈30 taxa) highlighted *accD* and *ycf1* as among the most informative coding-region hotspots and also reported that some medicinal mugwort taxa (e.g., *A. argyi*–*A. princeps*) can be nearly indistinguishable in plastome space, complicating chloroplast-only identification (Jiao et al., 2025). The complete chloroplast genome of *Artemisia hallaisanensis*, an endemic medicinal *Artemisia* from Korea (Jeju). They reported a 151,015-bp plastome with 80 protein-coding genes, 30 tRNAs, and four rRNAs, consistent with the typical plastome structure. Plastome phylogeny placed *A. hallaisanensis* close to *A. capillaris*, providing a Korea-endemic reference for future comparative evolution and authentication work (Lim et al., 2018). In another investigation, the complete chloroplast genome of *A. littoricola* specifically from Dokdo Island, Korea, was analysed for comparative and phylogenetic analyses within *Artemisia*. The study provided a Dokdo-anchored plastome reference useful for Korean biogeography, provenance tracing, and future evolutionary studies (Kadam et al., 2024).

### 4.6 Evolutionary basis of phytochemical diversification

Plastome phylogenies resolve maternal relationships but rarely explain specialized-metabolite diversification. Most specialized pathways depend on nuclear enzymes, regulators, and tissue-specific expression. Gene duplication and neofunctionalization can generate lineage-specific chemical profiles. In *A. argyi*, whole-genome duplication expanded terpene-synthase and secondary-metabolism gene families. These expansions contributed to volatile terpenoid diversity (Miao et al., 2022; Chen et al., 2023). AarADS produces α-bisabolol, unlike the *A. annua* ortholog producing amorpha-4,11-diene. This divergence demonstrates lineage-specific enzyme neofunctionalization (Chen et al., 2025).

In *A. annua*, ADS, CYP71AV1, DBR2, and ALDH1 mediate successive artemisinin-pathway steps (Teoh et al., 2006; Teoh et al., 2009). Trichome-specific regulators coordinate pathway expression, glandular development, and artemisinin accumulation (Teoh et al., 2006; Yuan et al., 2024). Comparative multi-omics also reveals chemical divergence among related taxa. *Artemisia stolonifera* showed sesquiterpenoid enrichment, whereas *A. argyi* showed monoterpenoid and flavonoid enrichment (Yuan et al., 2024). These differences corresponded with contrasting biosynthetic-gene expression (Cao et al., 2025).

Chemical profiles also reflect environmental and developmental plasticity. *Artemisia argyi* cultivars differed substantially in flavonoids and biosynthetic-gene expression (Qiao et al., 2023). UV-B exposure and nitrogen availability altered flavonoid and volatile accumulation (Gu et al., 2024). Therefore, phylogenetic proximity cannot independently predict phytochemistry or pharmacological activity. Within reviewed literature, direct evidence from Korean accessions remains limited. Whole plastomes provide stronger resolution than many single-locus chloroplast markers. Yet plastomes alone may not resolve hybridization, introgression, or recent divergence. Regulatory applications require population validation and integration with nuclear and chemical markers (Guo et al., 2025).

## 5 Phytochemical landscapes and ethnomedicinal use

The genus *Artemisia* is renowned for its rich phytochemical diversity, which has been a key factor in its widespread medicinal applications. Korean *Artemisia* taxa contain diverse metabolites (Flavonoids, Phenolic compounds, Alkaloids, Terpenoids) with reported biological activities. However, compound occurrence alone does not establish efficacy, exposure, or safety. Evidence was interpreted across four levels: ethnomedicinal, *in vitro*, animal, and clinical. Ethnomedicinal use documents historical practice but does not establish therapeutic efficacy. *In vitro* studies indicate biological plausibility under controlled experimental conditions. Animal studies provide organism-level evidence but may not predict human responses. Clinical efficacy requires reproducible benefits from adequately controlled human studies. Commercial availability was not considered evidence of safety or effectiveness (Berg et al., 2025).

Major advances include identifying species-associated metabolites and plausible molecular mechanisms. However, most activities derive from heterogeneous extracts and preclinical models. Standardized preparations, exposure data, and controlled human studies remain essential (Sharifi-Rad et al., 2022).

### 5.1 Secondary metabolite classes in *Artemisia*

#### 5.1.1 Terpenoids

Volatile terpenoids reported from Korean *Artemisia* include thujone, camphor, 1,8-cineole (Eucalyptol), α-terpineol, borneol, and camphene. Their abundance varies among species, organs, chemotypes, and extraction methods (Abad et al., 2012). More recent essential-oil profiling in *A*. *argyi* similarly identifies monoterpene-rich chemotypes dominated by 1,8-cineole, with additional contributions from thujone, terpinen-4-ol, and camphor, reinforcing the prevalence of monoterpenoids in Korean/East Asian *A. argyi* products (Li et al., 2024). Beyond volatiles, Korean native *A. iwayomogi* is notable for abundant sesquiterpene lactones (guaianolide-type terpenoids): isolation work from aerial parts reported new iwayomogins A and B together with numerous known sesquiterpene lactones, demonstrating that terpenoid evolution in Korean *Artemisia* extends well beyond essential-oil monoterpenes into structurally complex lactone scaffolds often linked to bioactivity (Son et al., 2022).

#### 5.1.2 Flavonoids

Flavonoids are another prominent and diverse class of secondary metabolites found in *Artemisia* species and are characterised by a typical Three-ring system (A, B, and C) (Falcone Ferreyra et al., 2012) (Figure 2). Flavonoids show antioxidant and anti-inflammatory effects across experimental models. Most evidence remains preclinical and depends on structure, concentration, and assay conditions (Min et al., 2009; Panche et al., 2016). Various classes, including flavones, flavonols, flavanols, anthocyanidins, and methoxyflavones, have been reported (Kim H. H. et al., 2020). Some common compounds included methoxyflavones such as eupatilin and jaceosidin, flavonols, for instance, e.g., quercetin-, kaempferol-, and rhamnetin-type compounds, the anthocyanidin-related compounds, C-glycosyl flavones, and biflavonoid-like structures; importantly, flavonoid profiles are strongly influenced by species identity, plant organ, and extraction/analytical conditions (Min et al., 2009; Kim S. M. et al., 2019; Kim H. H. et al., 2020).

**FIGURE 2:**
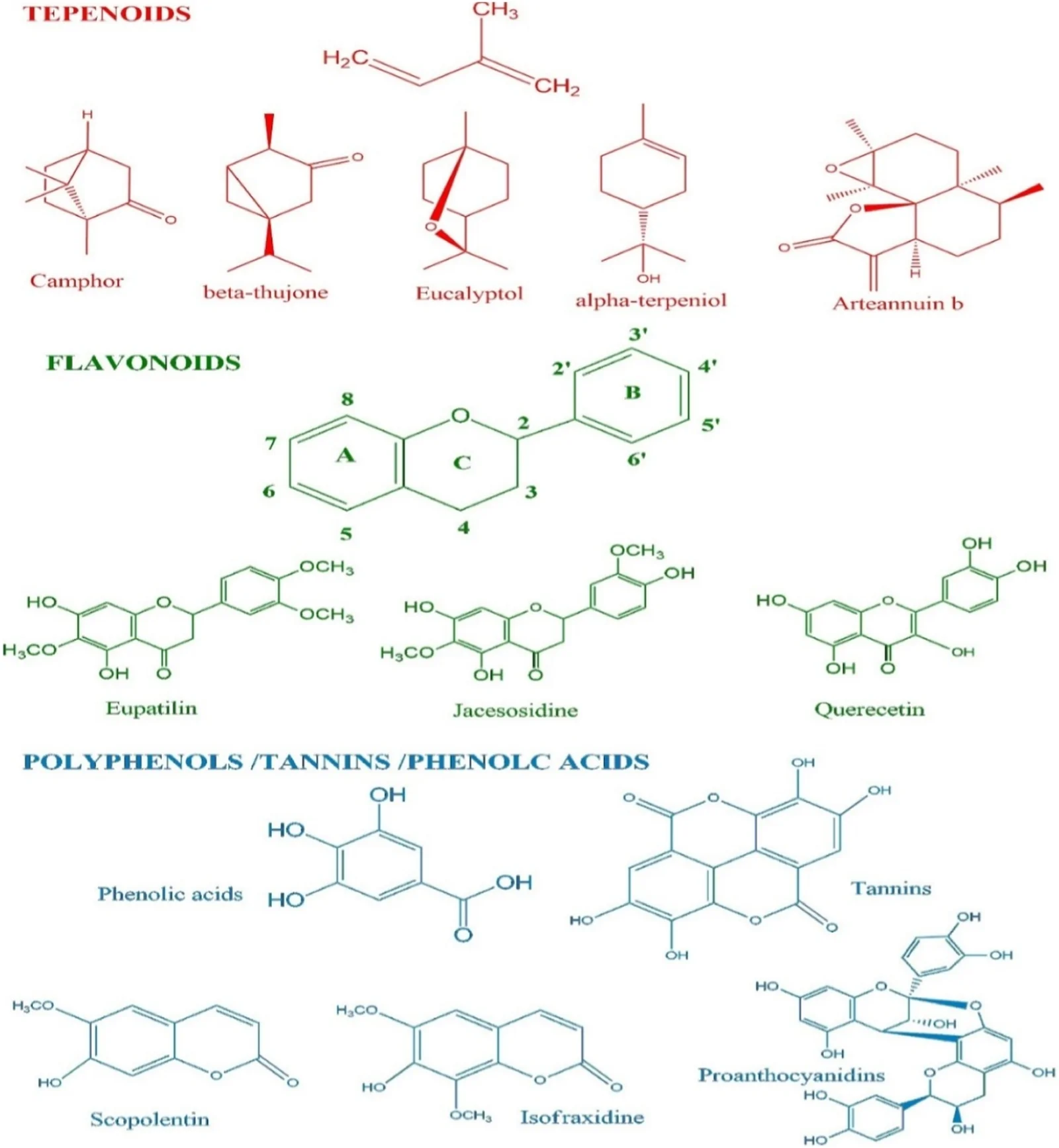
Representative phytochemical classes reported from Korean *Artemisia* taxa. Structures include terpenoids, flavonoids, phenolic acids, and tannin-related polyphenols. Compound occurrence varies by species, organ, chemotype, and extraction method. Structures do not indicate abundance, bioavailability, or clinical efficacy.

#### 5.1.3 Phenolic acids

Phenolic acids show redox and anti-inflammatory activity in experimental systems. Their clinical relevance remains limited by variable absorption and extensive metabolism (Zhang et al., 2012; Kim and Kim, 2021; Ekiert et al., 2022; Gu et al., 2023). The most prominent phenolic acids include hydroxycinnamic acid derivatives, especially caffeic acid, chlorogenic acid, ferulic acid, vanillic acid, gallic acid, Hydroxycinnamate glycosides and caffeoylquinic acids, including 3-O-, 4-O-, and 5-O-caffeoylquinic acid and coumarins like scopoletin and isofraxidin (Lee J. et al., 2017; Kim S. M. et al., 2019; Park M. H. et al., 2019; Kim and Kim, 2021).

#### 5.1.4 Tannins/polyphenolic compounds

Tannin-rich fractions show several experimental bioactivities. However, limited chemical characterization often prevents compound-level attribution (Song et al., 2016; Olennikov et al., 2018). In Korean *Artemisia* species, several tannins and polyphenolic compounds have been reported, including proanthocyanins and caffetannins (caffeoylquinic acid derivatives), as well as scoparone caffeoylquinic acids (Kimura et al., 1985; Toda, 2004; Song et al., 2016) (Table 3).

**TABLE 3:** Phytochemical constituents, reported biological activities, and evidence levels for *Artemisia taxa* recorded in Korea.

#	Species name	Major chemical compounds^a^	Reported activity	References
1	*A. annua L.*	Artemisinin, flavonoids, coumarins	Antimalarial, Antioxidant, Anti-inflammatory	(Kim et al., 2015b)
2	*A. iwayomogi*	Flavonoids, essential oils (e.g., thujone)	Against anemia Antibacterial, Muscle relaxant, AGEs inhibition	(Chan et al., 2002; Lee et al., 2017b)
3	*A. argyi H.*	Flavonoids, coumarins, tannins	Anti-inflammatory, Antioxidant, antidiabetic, Antimicrobial, cytotoxic	(Hwang et al., 2014)
4	*A. fukudo Makino*	Flavonoids, polyphenols	cytotoxic, Antimicrobial, Anti-inflammatory	(Kim et al., 2017; Kang et al., 2024)
6	*Artemisia capillaris*	Flavonoids, polysaccharides, essential oils (e.g., 1,8-cineole)	hepatoprotective, Antioxidant, Anti-inflammatory, Antiobesty	(Kim et al., 2010; Abad et al., 2012)
6	*A. japonica*	Flavonoids, coumarins, volatile oils (e.g., camphor)	Fever, Eczema, Antibacterial	(Kwon and Lee, 2001)
7	*A. vulgaris L.*	Flavonoids, volatile oils, sesquiterpene lactones	Digestive aid, Antibacterial, Antiviral, Sedative	(Abad et al., 2012)
8	*A. montana*	Flavonoids, triterpenoids, volatile Oils (e.g., thujone)	Heathherapy, Antioxidant, Antimicrobial, antiinflammatory	(Jeong et al., 2018)
9	*A. selengensis*	Flavonoids, triterpenoids	Dysmenorrhea, Anti-inflammatory, Hepatoprotective	(Kim et al., 2015a)
10	*A. princeps Pamp.*	Flavonoids, essential oils (e.g., camphor)	Antioxidant, Anti-inflammatory	(Hong et al., 2007)
11	*A. scoparia*	Flavonoids, triterpenes	Diuretic, Detoxifying, Antioxidant	(Yoon et al., 2006)
12	*A. sieversiana*	Triterpenes, flavonoids	Anti-inflammatory, Digestive aid, Antioxidant, Anthelminthic	(Nuermaimaiti et al., 2021)
13	*A. littoricola*	Flavonoids, triterpenes	Antimicrobial, Digestive aid, Anti-inflammatory	(Abad et al., 2012; Karadeniz et al., 2025)
14	*A. parvifolia Buch.-Ham*	Polyphenols, flavonoids	Antioxidant, Hepatoprotective, Anti-inflammatory, cytotoxic	(Karadeniz et al., 2025)
15	*A. sacrorum Ledeb*	Flavonoids, essential oils (e.g., thujone)	Anti-inflammatory, Antioxidant	(Yuan and Piao, 2011)
16	*A. stolonifera*	Flavonoids, sesquiterpenes	Anti-inflammatory, Digestive aid, Detoxifying	(Choi et al., 2013)
17	*A. gmelinii*	Essential oils (e.g., linalool), flavonoids	Antioxidant, Antiobesity, Antimicrobial, Digestive aid, Antifungal	(Park et al., 2019b)
18	*A. apiacea*	Flavonoids, coumarins, triterpenes	Detoxifying, Anti-inflammatory, Hepatoprotective	(Abad et al., 2012)
19	*A. keiskeana*	Flavonoids, essential oils	Antimicrobial, Anti-inflammatory, Hepatoprotective	(Choi et al., 2013)
20	*A. indica*	Artemisinin, flavonoids, triterpenoids	Anti-malarial, Anti-inflammatory, Antioxidant	(Abad et al., 2012; Ruwali et al., 2017)
21	*A. rubripes* Nakai	Flavonoids, volatile oils (e.g., camphor)	Anti-inflammatory, Antioxidant	(Lee et al., 2014)
22	*A. lancea* *Vaniot*	Flavonoids, essential oils (e.g., 1,8-cineole)	Antioxidant, Anti-inflammatory	(Abad et al., 2012)
23	*A. dubia*	Flavonoids, essential oils	hepatoprotective, Anti-inflammatory, Cancer, Antioxidant	(Lee et al., 2018b)
24	*A. hallaisanensis*	Flavonoids, triterpenes	Antioxidant, Anti-inflammatory, Respiratory problems	(Lim et al., 2018; Kim and Kim, 2021)

^a^ Evidence levels refer to the cited studies. *In vitro* and animal findings indicate preclinical activity and should not be interpreted as zestablished clinical efficacy.

#### 5.1.5 Mechanistic and translational interpretation

Sesquiterpene lactones often contain electrophilic α-methylene-γ-lactone groups. These groups modify nucleophilic protein residues through Michael addition. This reactivity may suppress inflammatory signaling, including NF-κB. However, broad protein reactivity can reduce target selectivity (Emami et al., 2010; Ivănescu et al., 2015). Flavones, including eupatilin and jaceosidin, modulate NF-κB, MAPK, and Nrf2 pathways. These mechanisms derive mainly from cellular and animal studies (Shin et al., 2022).

Hydroxylation, methoxylation, and glycosylation influence target binding, permeability, metabolism, and bioavailability (Billowria et al., 2022). Chlorogenic acids undergo extensive intestinal and microbial metabolism. Consequently, circulating metabolites may differ substantially from tested parent compounds (Clifford et al., 2020). Similarly, Eupatilin and jaceosidin inhibit CYP1A2 and CYP2C9 under *in vitro* conditions. Their interaction potential therefore requires clinical pharmacokinetic evaluation (Ji et al., 2010). Translation requires standardized extracts, exposure data, toxicity studies, and controlled clinical trials.

Extraction procedures, concentrations, controls, and assay conditions differ among the cited studies. Therefore, reported activities should not be compared directly across preparations. Complete methodological details are available in the respective primary studies. Extract composition and bioactivity depend strongly on preparation and chemical characterization (Heinrich et al., 2022). Reported activities do not establish clinical efficacy.

### 5.2 Historical, ethnopharmacological, and regulatory context

In Korea, the traditional uses of *Artemisia* are best understood through pharmacognostic categories used in traditional Korean medicine (TKM) rather than species names alone. The most important categories are Aeyup (애엽; mugwort leaf drug) and Injinho/Injinho (인진호/인진; capillary wormwood-related drug), with Haninjin (한인진) recognized separately. Molecular authentication studies repeatedly emphasize that these categories are therapeutically distinct but morphologically confusable in dried herbal markets, which has practical implications for quality control, efficacy, and safety (Lee et al., 2006) (Figure 3; Table 4).

**FIGURE 3:**
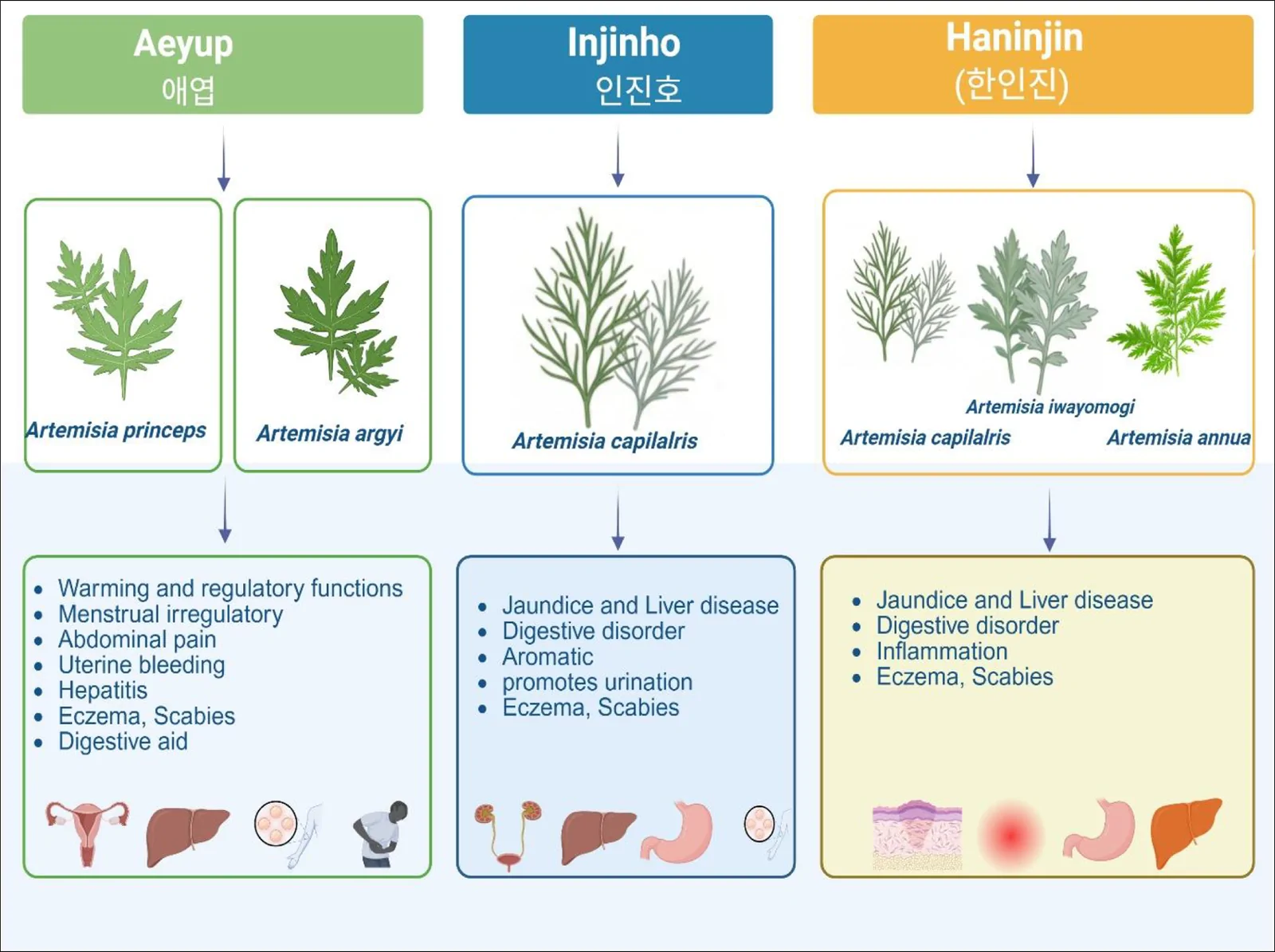
Principal ethnopharmacological categories of *Artemisia* drugs in traditional Korean medicine. *Aeyup* includes *Artemisia princeps* and *Artemisia argyi*. *Injinho* primarily denotes *Artemisia capillaris*. *Haninjin* denotes *Artemisia iwayomogi*. *In-Jin-Sook* may contain several taxa, depending on historical practice. These categories require botanical authentication and should not be considered interchangeable.

**TABLE 4:** Ethnopharmacological identity, evidence context, and regulatory priorities of Korean *Artemisia* drugs.

Korean drug category	Principal taxa	Evidence context	Regulatory priority	References
*Aeyup*	*A. princeps*; *A. argyi*	Traditional leaf drug; preclinical anti-inflammatory evidence	Exclude *Injinho* and *Haninjin* substitution	(Lee et al., 2006)
*Injinho*	*A. capillaris*	Traditional medicinal drug; preclinical pharmacology	Confirm species and medicinal part	(Doh et al., 2016)
*Haninjin*	*A. iwayomogi*	Distinct Korean drug; preclinical antimicrobial evidence	Differentiate from *Injinho* and *Aeyup*	(Lee et al., 2008)
*In-Jin-Sook*	Several *Artemisia* taxa	Historical mixed preparation; limited formulation-specific evidence	Define species composition and preparation	(Ahn, 2000)

These Korean categories represent distinct pharmacognostic drugs, not interchangeable botanical synonyms. *Aeyup* includes *Artemisia princeps* and *A. argyi*, whereas *Injinho* denotes *A. capillaris*. *Haninjin* refers to *A. iwayomogi*. *In-Jin-Sook* historically includes several *Artemisia* taxa. Overlapping morphology creates substitution risks in dried herbal materials. SCAR and multiplex PCR assays can distinguish these medicinal categories reliably (Lee et al., 2008; Doh et al., 2016).

#### 5.2.1 *Artemisia princeps* (쑥) and the “Aeyup” tradition in Korea

*Korean mugwort* (*A*. *princeps*) is one of the most culturally and medically important *Artemisia* species in Korea including appearance in foundational Korean myth, and records both medicinal and folk/domestic uses (Lee et al., 2006). Traditionally uses include warming and regulating functions, applications for menstrual irregularity, abdominal cold pain, bleeding-related symptoms, chronic hepatitis-related symptoms, eczema/scabies-like conditions and digestive complaints in traditional practice (koreantk.com) (Lee et al., 2008).

#### 5.2.2 *Artemisia argyi* and overlap within the “Aeyup” drug concept

Although Korean practice commonly associates “ssuk” with *A. princeps*, pharmacognostic studies show that both *A. princeps* and *A. argyi* are grouped under Aeyup in Korean traditional medicine contexts. A landmark marker-development study (SCAR-based discrimination) explicitly states that *A. princeps* and *A. argyi* are classified as Aeyup and used as important medicinal materials in TKM (koreantk.com (Lee et al., 2008).

#### 5.2.3 *Artemisia capillaris* (사철쑥; Injinho/Injin)

Among Korean *Artemisia* taxa, *A. capillaris* has the strongest and most consistent association with Injin/Injinho (茵蔯), a traditional remedy used for jaundice and hepatobiliary/liver-related conditions. The Korean Traditional Knowledge Portal entry for *A*. *Capillaris* Herba (Injin) lists the aerial part as the medicinal material. It describes traditional efficacy concepts linked to jaundice relief, digestive disorders, clearing “liver heat,” and damp-heat removal with facilitation of urination (koreantk.com (Lee et al., 2008).

#### 5.2.4 *Artemisia iwayomogi* (더위지기; Haninjin)

*Artemisia iwayomogi* (Haninjin) is recognised in Korea as a distinct medicinal *Artemisia*, not merely a synonym of Injinho/Aeyup (Ju et al., 2021). More recent phytochemical work on *A. iwayomogi* also preserves the ethnomedicinal context, noting that in Korea, the aerial parts of *A. iwayomogi* (Haninjin) have long been used as a replacement for *A. capillaris* since both herbs have been used in inflammatory/liver/skin-related conditions, including jaundice, fever, pruritus, and liver cirrhosis contexts (koreantk.com (Son et al., 2022).

#### 5.2.5 *Artemisia capillaris, Artemisia iwayomogi, and Artemisia annua* “(인진석)In-Jin-Sook

The traditional Korean medicine In-Jin-Sook is largely composed of at least three *Artemisia* species, including A*. capillaris, A. iwayomogi, and A. annua*, which have been commonly used (often as long-boiled water extracts of dried stems/leaves) for liver disease-related purposes (Ahn, 2000).

Traditional indications provide historical context but do not establish clinical efficacy. *Artemisia capillaris* and *A. iwayomogi* show distinct chemical profiles and antimicrobial activities (Seo et al., 2010). These differences may affect product composition, biological response, and batch consistency. Regulatory standards should define accepted species, plant parts, markers, and substitution limits. DNA authentication should complement microscopy and chromatographic fingerprinting. This orthogonal strategy strengthens identity, traceability, and quality control (Abubakar et al., 2017; Nazar et al., 2024).

## 6 Commercial and therapeutic applications

With reference to traditional uses in Korea, there exists an immense demand for herbal products comprising *Artemisia* species, especially the most prominent species, including *A*. *annua, A*. *argyi and A*. *iwayomogi*. This section explores the commercial applications of *Artemisia* species, focusing on their growing roles in pharmaceuticals, cosmeceuticals.

### 6.1 Cosmeceuticals and biotechnology products

Various *Artemisia* extracts are being used in the cosmeceutical industry (Ekiert et al., 2022), like diverse skin care products that employ the use of *A. argyi* as a key ingredient due to its anti-inflammatory and antioxidant properties (Wang et al., 2023b). The flavonoids and essential oils present in *A. argyi* are known to protect the skin from oxidative stress and UV-induced damage (Lee and Lim, 2020). Studies have shown that these compounds can reduce collagen degradation and prevent skin ageing, positioning *A. argyi* as a candidate botanical ingredient in anti-ageing products (Li et al., 2024). In addition, *A*. *iwayomogi* is valued in cosmetics for its antimicrobial and anti-inflammatory properties, especially in the treatment of acne and rosacea (Kim K. Y. et al., 2019; Park D. et al., 2019; Jenis et al., 2025).

The strongest mechanistic rationale for cosmetic use currently lies in the anti-inflammatory/skin-soothing domain, which is highly relevant to redness-prone and barrier-stressed skin. Experimental evidence supports the anti-inflammatory activity of *A. princeps* on skin by suppressing inflammasome activation (NLRP3 and AIM2) *via* inhibition of ASC phosphorylation (Kwak et al., 2018). Additional evidence from Korean-associated research contexts indicates anti-inflammatory effects of *Artemisia* leaf extracts (including *A. princeps* and *A. argyi*) in contact dermatitis models, with reported reductions in ear swelling, inflammatory cytokines, and COX-2/iNOS-linked products after topical administration in mice (Kim, 2023). Likewise, *Artemisia apiacea* extract demonstrated improvement in atopic dermatitis-like skin lesions *in vivo* and suppression of pro-inflammatory chemokines in TNF-α/IFN-γ-stimulated keratinocyte models, further reinforcing the relevance of *Artemisia* taxa to soothing and anti-irritation cosmetic concepts (Yang et al., 2018). The data in Table 3 briefly describes the commercial application of Korean products containing various *Artemisia* species (Table 5).

**TABLE 5:** Illustrative Korean skincare products declaring *Artemisia*-derived ingredients and their manufacturer-reported claims.

Product	Product type	*Declared Artemisia* ingredient	Manufacturer claim	Source
HANYUL *Artemisia* Soothing Toner	Skincare toner	*A*. *argyi* extract	Calms and hydrates sensitive skin	HANYUL Global (Olive Young)
HANYUL Pure *Artemisia* Cleansing Pack Foam	Facial cleanser	*Artemisia* extract	Gentle daily cleansing and soothing	HANYUL Global (Olive Young)
OFHAN *Artemisia* Soothing Serum	Serum	*Artemisia* extract	Soothing effect	OFHAN
Isntree Mugwort Soothing Serum	Serum	Mugwort extract (*Artemisia* spp.)	Kin regeneration	Isntree Official
I’M FROM Mugwort Cream	Moisturizer	*Artemisia princeps extract*	Deep hydration	Olive young
MISSHA *Artemisia* Calming Ampoule Pads	Skincare pads	*Artemisia* extract	Moisturizer	MISSHA
ROUND LAB Mugwort Calming Sheet Mask	Sheet mask	*Artemisia* extract	Anti-inflammatory	Olive young
HANYUL *Artemisia* Cica Repair Mask	Sheet mask	*Artemisia vulgaris* extract	Relieves redness	HANYUL Global Olive young

Current evidence for topical *Artemisia* products remains predominantly preclinical. Mechanistic studies report reduced inflammasome, NF-κB, MAPK, STAT1, and chemokine signaling. *Artemisia princeps* suppressed NLRP3 and AIM2 activation through reduced ASC phosphorylation. However, this evidence used macrophages and a mouse peritonitis model (Kwak et al., 2018). *A*. *apiacea* reduced inflammatory chemokines in keratinocytes and dermatitis severity in mice (Yang et al., 2018). *Artemisia argyi* also improved dermatitis-like lesions through several inflammatory pathways (Han et al., 2016). These findings support biological plausibility, but not consumer-level efficacy. Controlled human trials remain scarce for Korean *Artemisia* formulations.

Translation requires standardized extracts, marker compounds, dermal exposure, and sensitization testing. Formulations must address oxidation, solubility, penetration, preservative compatibility, and batch variability. Delivery systems may improve stability and topical bioavailability. However, formulation benefits require product-specific clinical confirmation (Omidian et al., 2026). Korean functional cosmetics require MFDS evaluation or product reporting. Submissions must address product safety and effectiveness. Disease-oriented wording should not replace permitted cosmetic claims. Commercial claims must therefore remain separate from preclinical pharmacological evidence.

Products were purposively selected as illustrative market examples if they were marketed by Korean skincare brands and declared an *Artemisia*-derived ingredient on an official manufacturer or authorized retailer website. This selection was not systematic or exhaustive. Species names were recorded only when explicitly stated by the manufacturer; otherwise, the ingredient was reported as *Artemisia* spp. Retail claims were treated as manufacturer-reported statements and not as evidence of independently verified efficacy or regulatory approval.

Commercial availability demonstrates market interest, not product-specific efficacy. Mechanistic plausibility remains insufficient without formulation stability, exposure assessment, and human validation. Future studies should evaluate standardized products using predefined clinical and safety outcomes (Ekiert et al., 2022).

## 7 Regulatory, quality, and safety considerations

The marketing of herbal products needs scientific safety data as suggested by Regulatory agencies including U.S. Food and Drug Administration (FDA) and the European Medicines Agency (EMA) (Rahman et al., 2025). These data must be supported with detailed toxicity and safety dosage recommendations and approved clinical trials (Gherghescu and Delgado-Charro, 2020). Due to the complex nature of herbal extracts, the Quality control (QC) is a crucial factor for consideration in the commercialization process, and data must be supported with advanced high-performance liquid chromatography (HPLC) and gas chromatography-mass spectrometry (GC-MS), *etc.* (Mishra et al., 2018). Furthermore, in order to maintain high product standards and product consistency, the development of Good Manufacturing Practices (GMP) for herbal products [157] such as *Artemisia*-derived products is of prime importance.

Standardization should combine botanical authentication, chromatographic fingerprints, and quantitative marker analysis. Single-marker assays cannot capture chemotype, harvest, extraction, and batch variability (Noviana et al., 2022). Specifications should also cover pesticides, heavy metals, microbes, residual solvents, and adulterants. Validated HPLC and GC–MS methods should confirm identity, content ranges, and batch consistency (Ichim et al., 2022).

Traditional use does not establish safety across species, preparations, doses, or exposure durations. Safety conclusions must therefore remain species-specific and preparation-specific (Höld et al., 2000). Thujone-containing oils require caution because α-thujone modulates GABAA_AA receptor signaling (Lachenmeier, 2010). High exposure can cause neuroexcitation and seizures. However, thujone levels vary across species, organs, chemotypes, and commercial products. Findings from *Artemisia absinthium* cannot be generalized across Korean *Artemisia* taxa (Di Lorenzo et al., 2018).

Human hepatotoxicity evidence for Korean *Artemisia* preparations remains sparse and causally uncertain. Therefore, genus-wide liver-damage claims should be avoided. Suspected injury requires alternative-cause exclusion and structured causality assessment (Stickel and Shouval, 2015).

*In vitro* studies indicate CYP inhibition by selected *Artemisia* extracts and constituents. Eupatilin and jaceosidin inhibit CYP1A2 and CYP2C9 experimentally. *Artemisia annua* extracts also inhibit CYP2B6 and CYP3A4 *in vitro*. Clinical relevance remains uncertain because formulations and systemic exposures differ (Kondža et al., 2023). Adverse-event reports should record species, plant part, preparation, dose, duration, and indication. Batch number, co-medications, latency, dechallenge, rechallenge, and laboratory findings should also be documented. Serious events require regulatory reporting and retained product samples. This traceability supports causality assessment and corrective quality investigations (Frenzel and Teschke, 2016). Labels should specify dosage, contraindications, interaction warnings, storage conditions, and adverse-event reporting pathways (World Health, 2004; Tsai et al., 2012).

Analytical standardization remains inconsistently applied across *Artemisia* products. Safety conclusions remain uncertain because interactions and adverse events are poorly documented. Harmonized specifications and pharmacovigilance systems should support regulatory decisions (Noviana et al., 2022).

## 8 Challenges, future directions, and sustainability

Korean demand for *Artemisia*-based products requires reliable cultivation, authenticated germplasm, and consistent phytochemical quality. Sustainable cultivation of *Artemisia* species is crucial due to the increasing demand for bioactive compounds (Negri et al., 2024). Key strategies like “monocropping” can lead to water scarcity, soil depletion and loss of biodiversity. Here the sustainable farming practices can be adopted to ensure long term availability of *Artemisia*. The integrated pest management, use of organic fertilisers and substancial crop rotation can be helpful in mitigating environmental damage (Ali et al., 2017). Initial cultivation evidence exists for selected Korean *Artemisia* taxa. Controlled studies defined suitable germination temperatures and storage conditions for *Artemisia dubia*. However, comparable protocols remain unavailable for most Korean taxa. Future studies should prioritize authenticated accessions, multisite cultivation, yield stability, and chemotype consistency (Kim et al., 2021).

Transcriptomics and metabolomics already support pathway discovery within selected *Artemisia* species. However, evidence remains concentrated in *A. annua*, *A. argyi*, and several studied congeners. Korean taxa require voucher-linked populations, matched metabolomes, and environmental metadata. Multi-omics should test, rather than assume, genotype–metabolite relationships (Yang et al., 2022; Yang et al., 2022) CRISPR remains an emerging option for Korean *Artemisia* taxa. Candidate metabolic regulators have been identified in *A. annua*. However, Korean transformation systems and field performance remain insufficiently validated. Editing should follow functional validation, transformation optimization, and regulatory assessment (Shen et al., 2022).

Combination therapies remain hypothetical for most Korean *Artemisia* preparations. Preliminary *A. annua* evidence suggests interactions among extract constituents. However, clinical relevance remains uncertain. Future studies require standardized preparations, pharmacokinetics, interaction testing, and quantitative synergy analysis (Rasoanaivo et al., 2011) (Figure 4).

**FIGURE 4:**
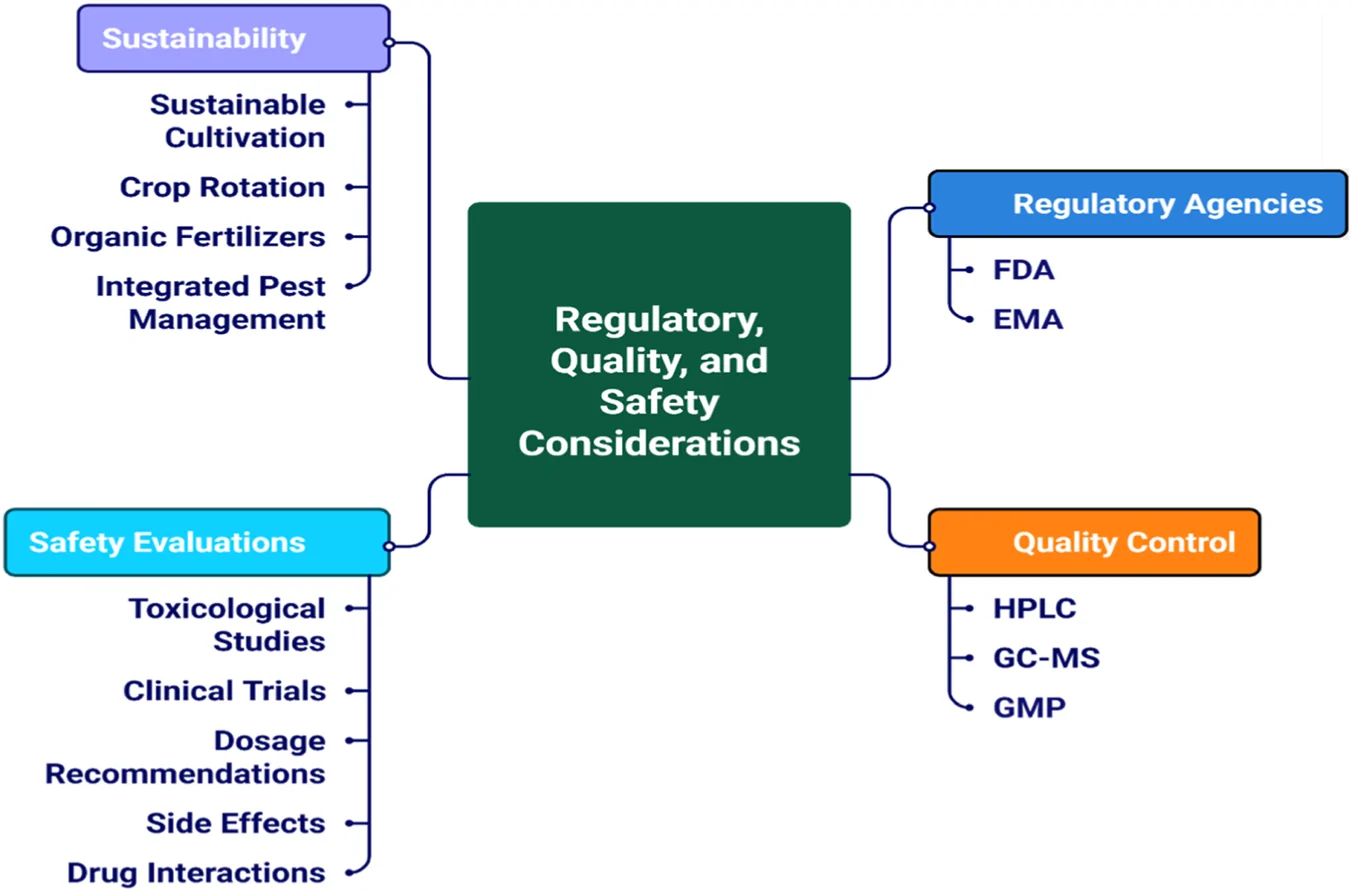
Integrated framework for sustainable and quality-controlled *Artemisia* product development. The framework links cultivation, authentication, phytochemical standardization, safety assessment, and regulatory oversight. Multi-omics and genome editing represent emerging research tools. These approaches remain investigational for most Korean *Artemisia* taxa.

## 9 Conclusion

The plants of genus *Artemisia* are considered as noteworthy and multifaceted resource that contribute profound values to both traditional and contemporary medicine. Key Korean native *Artemisia* species have been provided with promising medicinal potential across various applications, especially in cosmetics. Korean *Artemisia* extracts show diverse biological activities in experimental studies. However, clinical efficacy remains insufficiently established for most preparations. Such key compounds are mainly responsible for significant anti-inflammatory, antimicrobial, antioxidant activities of *Artemisia* based products. The Cp genomes advances has enabled scientist to the evolutionary relationships among *Artemisia* species, thereby providing crucial insights into their genetic diversity, adaptation, and geographic distribution. Through a comparative plastome analysis the identification of key markers for *Artemisia* species differentiation and informed cultivation and conservation strategies is possible. A clear understanding of molecular signaling pathways and molecular targets of these *Artemisia* bioactive components is crucial for development of novel therapeutic agents. Despite its immense commercial potential, key *Artemisia plants* face several challenges in sustainable cultivation, regulatory standardization, and quality control. An adaptation of sustainable farming protocol is required to meet the global demand of *Artemisia*-based. Also and key regulatory framework is proposed to ensure the safety, efficacy and consistency of *Artemisia*-based products, thereby facilitating their broader acceptance in clinical and commercial settings.
