# Supplementary material for: Korean Artemisia species: phylogenomic authentication, ethnopharmacology, and commercial applications
Source: Front Pharmacol. 2026 Sep 16;17:1873437. doi: 10.3389/fphar.2026.1873437 (PMC13624125; doi:10.3389/fphar.2026.1873437)
Supplement: Supplementary file 1 [file DataSheet1.pdf]

Supplementary data

Korean *Artemisia* Species: Phylogenomic Authentication, Ethnopharmacology, and Commercial Applications

Adnan Amin[1*], Khizar Abbas[2] and Mozaniel Santana de Oliveira[3]

[1]Department of Life Sciences, Yeungnam University, Gyeongsan 38541, Republic of Korea.
[2] School of Pharmacy, University College London, United Kingdom. khizar.abbas@ucl.ac.uk

[3] Laboratory of Pharmacology of Inflammation and Behavior, Graduate Program in Pharmaceutical Sciences, Institute of Health Sciences, Federal University of Pará, Belém, 66075110, Brazil. mozaniel@ufpa.br

Correspondence

Adnan.amin@yu.ac.kr

Figure S1 summarizes plastome relationships among representative *Artemisia* taxa sampled in Korea. The topology follows 80 plastome protein-coding genes (Kim et al., 2020). Five major maternal lineages were recovered. *A. capillaris*, *A. hallaisanensis*, and *A. japonica* occurred within subgenus *Dracunculus*. *A. gmelinii* belonged to a separate plastome lineage. Near-identical plastomes limited discrimination among several closely related medicinal taxa. These patterns may reflect recent divergence, hybridization, or chloroplast introgression. Plastome relationships should therefore be interpreted alongside nuclear genomic evidence (Jiao et al., 2023). *A. littoricola* is presented separately because its placement derives from an independent dataset.

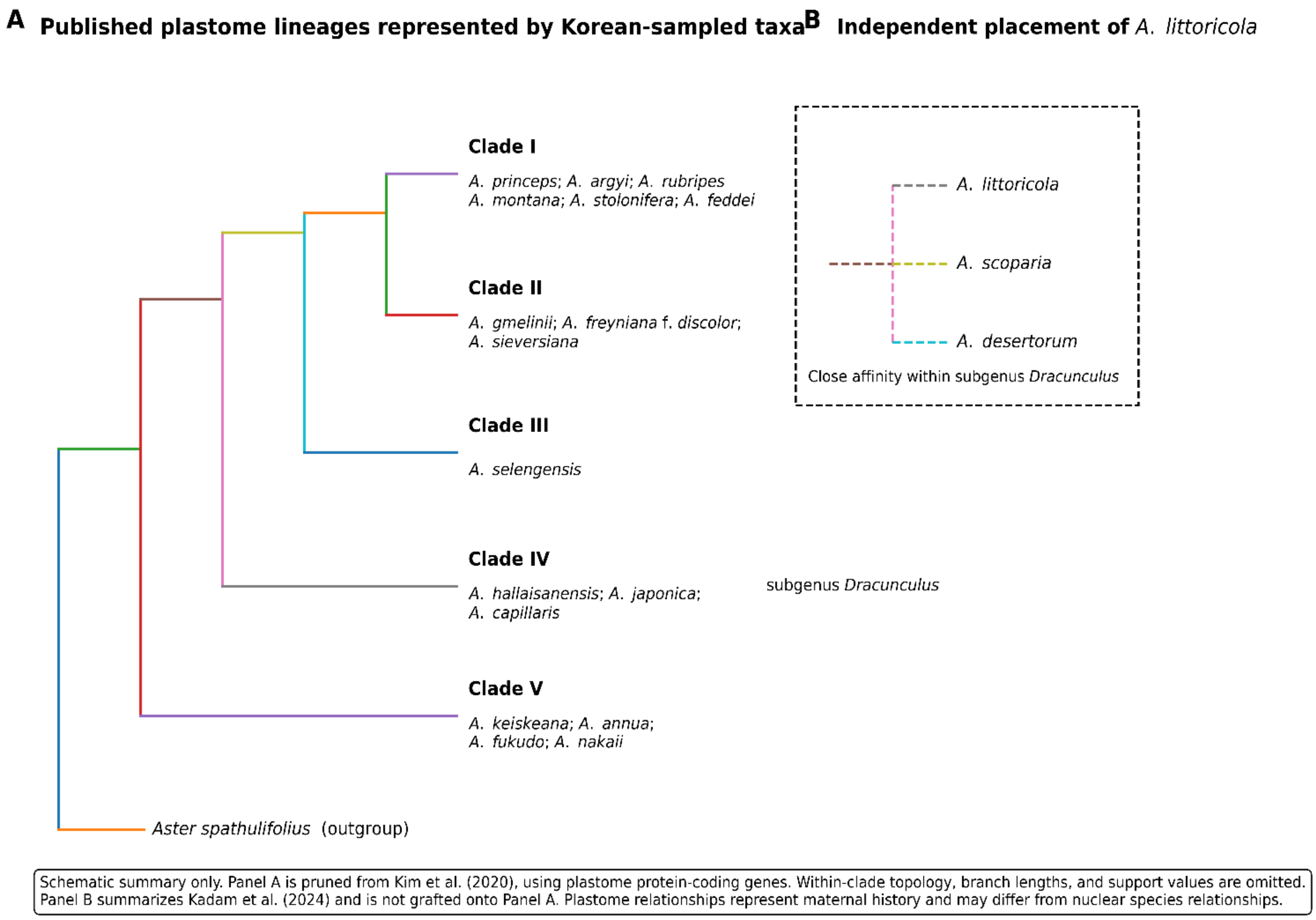

Figure X. Simplified plastome cladogram of representative *Artemisia* taxa sampled in Korea. Panel A presents a pruned schematic of the topology reported by Kim et al. (2020). Their phylogenetic reconstruction used 80 non-redundant plastome protein-coding genes. Duplicate accessions and external reference taxa were removed for clarity. Clade membership follows the published Bayesian and maximum-likelihood analyses. Within-clade relationships, branch lengths, and support values are not reproduced. Panel B summarizes the independent placement of *A. littoricola* reported by Kadam et

al. (2024). That species showed close affinity with *A. scoparia* and *A. desertorum* within subgenus *Dracunculus*. Panel B is not grafted onto Panel A. Plastome phylogenies represent maternal evolutionary history and may differ from nuclear species relationships. Panel A was adapted from Kim et al. (2020) under the Creative Commons Attribution 4.0 License.

References

Jiao, B., Chen, C., Wei, M.-Y., Niu, G., Zheng, J., Zhang, G., Shen, J.-H., Vitales, D., Vallès, J., Verloove, F., Erst, A., Soejima, A., Mehregan, I., Kokubugata, G., Chung, G., Ge, X., Gao, L., Yuan, Y., Joly, C., Jabbour, F., Wang, W., Shultz, L., and Gao, T.-G. (2023). Phylogenomics and morphological evolution of the mega-diverse genus Artemisia (Asteraceae: Anthemideae): implications for its circumscription and infrageneric taxonomy. *Annals of Botany* 131, 867-883.

Kim, G.-B., Lim, C., Kim, J.-S., Kim, K., Lee, J., Yu, H.J., and Mun, J. (2020). Comparative chloroplast genome analysis of Artemisia (Asteraceae) in East Asia: insights into evolutionary divergence and phylogenomic implications. *BMC Genomics* 21.
